# Performing One-Session Cognitive Stimulation to Interact with Patients with Dementia in a Hospital for Mood Improvement: A Retrospective Single-Arm Cohort Study

**DOI:** 10.3390/ijerph19031431

**Published:** 2022-01-27

**Authors:** Kenji Tsuchiya, Miku Saito, Naoto Okonogi, Saori Takai, Yoko Jingu, Koji Tanaka, Kazuki Hirao, Takaaki Fujita, Yukiko Tanaka

**Affiliations:** 1 Department of Rehabilitation Sciences, Gunma University Graduate School of Health Sciences, 3-39-22, Showa, Maebashi 371-8514, Japan; kojit929@gunma-u.ac.jp (K.T.); kazuki.hirao@gunma-u.ac.jp (K.H.); 2 Department of Rehabilitation, Medical Corporation Taiseikai, Uchida Hospital, 345-1, Kuyaharamachi, Numata 378-0005, Japan; reha@taiseikai-group.com (M.S.); saori-takai@taiseikai-group.com (S.T.); y-jingu@taiseikai-group.com (Y.J.); y-tanaka@kyujinkai.com (Y.T.); 3 Tokyo Dementia Care Research and Training Center, 1-12-1, Takaidonishi, Suginamiku, Tokyo 168-0071, Japan; naoto-okonogi@taiseikai-group.com; 4 Department of Occupational Therapy, School of Health Sciences, Fukushima Medical University, 10-6 Sakaemachi, Fukushima City 960-8516, Japan; t-fujita@fmu.ac.jp

**Keywords:** dementia, hospital, mood, care, activity, rehabilitation

## Abstract

Developing support and an environment for patients with dementia in hospitals is important. This study aims to assess the immediate effect of one-session cognitive stimulation intervention on the mood of patients with dementia in a hospital as preliminary evidence. This study included 33 female patients. The cognitive stimulation intervention was conducted in the day room of the hospital ward by two occupational therapists. The patients participated in one or more sessions. The cognitive stimulation intervention was designed to discuss current affairs that implicitly stimulate memory, executive function, and language skills, according to the cognitive stimulation definition. Outcomes were evaluated using a two-dimensional mood scale. The primary outcome was pleasure. The before and after session scores for the first session and the average score before and after each session at multiple times were compared. The patients’ pleasure showed significant improvements in both analyses. These results may indicate that one-session Cognitive stimulation intervention in a hospital effectively improves a mood of pleasure immediately. This study is the first report to provide preliminary evidence on the beneficial alterations of mood after one-session cognitive stimulation intervention for patients with dementia in hospitals. Cognitive stimulation intervention may be an effective non-pharmacotherapy for these patients.

## 1. Introduction

In 2018, a total of 50 million patients with dementia were diagnosed, and these numbers are estimated to increase to 152 million by 2050 [1]. The number of fractured femurs, lower respiratory tract infections, urinary tract infections, and head injuries was higher in patients with dementia than in those without dementia [2]. Patients with dementia had a higher readmission rate within 30 days (7–35%) than those without dementia [3]. In addition, the length of hospital stay of patients with dementia was longer than that of those without dementia [4]. In a systematic review, the prevalence estimate for patients with dementia in hospitals was 12.9–63.0% [5]. These reports show that the number of patients with dementia will increase in hospitals.

Hospitals can be loud and have an unfamiliar environment, which can add to confusion and may trigger responsive behaviors in patients with dementia [6]. Responsive behaviors are yelling, hitting, restlessness, and repetitive questioning, which are words or actions that patients with dementia use to convey their needs. The estimated rate of responsive behaviors was approximately 75% in patients with dementia [6]. Hospitals are not equipped to provide the best care for patients with dementia [7]. Therefore, developing support and a friendly environment for patients with dementia in hospitals is important [8].

As pharmacological approaches for patients with dementia have limitations [9], nonpharmacological approaches are very important [10]. The advantage of nonpharmacological approaches is that they have minimal adverse side effects. Accordingly, these can be combined with other nonpharmacological approaches and pharmacological treatments without major concerns of interference [10]. Some international and large studies based on nonpharmacological approaches for patients with dementia concluded that cognitive stimulation (CS) is a highly recommended approach [11,12,13]. The definition of CS [14] is engagement in a range of activities and discussions including social interaction. The CS intervention is designed to discuss current affairs that implicitly stimulate memory, executive function, and language skills. This intervention addresses dysfunctional aspects caused by an inadequately stimulating and rewarding social environment. Thus, CS intervention aims for the general enhancement of cognitive and social functioning. The widely used CS program includes 14 sessions of themed activities, which are conducted twice weekly, and was developed in the United Kingdom [15]. A systematic review on the UK CS program showed improvements in quality of life, depression, and cognition [16]. However, participants in these studies were people with dementia living in community and residential care units, which are long-term admission facilities. In addition, these interventions focus on repetitive cumulative effects over several weeks. Because the hospitalization duration is short for patients with dementia, the immediate effect of a one-session intervention is important for determining clinical and methodological significance. However, whether CS interventions in hospitals can immediately improve the moods of patients with dementia is unclear. Patients with dementia admitted to hospitals feel stress and unpleasant moods due to the environmental load every day. The accumulative effect does not improve the unpleasant moods that these patients experience daily. In addition, an immediate improvement in pleasure may motivate patients with dementia to continue participation in CS intervention. The effects of one-session mood changes have been reported in many previous studies, such as music therapy, exercise, relaxation, and meditation [17,18,19,20]. In this study using retrospective single-arm cohort design, we hypothesize that one-session CS intervention has a beneficial effect on mood and assess its effectiveness. This study is important as preliminary evidence to consider methods of non-pharmacotherapeutic interventions for patients with dementia in hospitals.

## 2. Materials and Methods

### 2.1. Participants

This retrospective single-arm cohort study analyzed electronic medical records of 33 female patients (85.8 ± 7.8 years) treated at Uchida Hospital from October 2019 to March 2020. Calculating an appropriate sample size is an essential step before conducting a study, as samples that are too small or too large may result in type I (α) or II (β) errors, respectively. For this study, the type I error was fixed at a maximum value of 5%, the type II error was fixed at a maximum value of 20%, and the effect size was fixed as medium (r = 0.5) [21]. Using this method and calculating software (R2. 8. 1.), we determined that this study required a sample size of 33.4.

The following inclusion criteria were applied according to the previous CS research [22]: (1) patients participated in one or more CS sessions; (2) patients had to have been diagnosed with dementia in the mild-to-moderate range (a score of ≥10 on the Mini-Mental State Examination); and (3) the patient’s sight, hearing, and communication abilities were well enough to participate in the group. This study was approved by the Institutional Review Board of Uchida Hospital (No. 2020-006). Patient anonymity was preserved.

### 2.2. Intervention

The group CS intervention was conducted once a week for 1 h in the day room of the ward. All patients included in this study participated in at least one session. Some patients were able to participate in more than one session due to the length of their hospital stay. The outcomes of the participants were measured before and after each session. Two occupational therapists with sufficient clinical experience for patients with dementia supervised sessions of four or five participants. Various activities (body exercise, calendar creation using collage with traditional Japanese paper, singing, reminiscence, cooking, and reality orientation) were selected based on the patient’s physical function, cognitive function, and life history. The CS intervention was designed to discuss current affairs that implicitly stimulate memory, executive function, and language skills, according to the CS definition [14].

### 2.3. Outcome Measures

Mood changes before and after sessions were evaluated using the two-dimensional mood scale (TDMS), the Japanese version [19]. This questionnaire evaluates pleasure, arousal, vitality, and stability states. Each mood state in TDMS has been associated with a different mood factor and the scale is highly reliable in terms of internal consistency (Cronbach’s alpha of pleasure = 0.77, arousal = 0.58, vitality = 0.80, and stability = 0.83) [19]. The primary outcome was the pleasure state. The secondary outcomes were the arousal, vitality, and stability states. The TDMS comprises an eight-item self-administered questionnaire answered on a 6-point Likert-type scale: “Not at all” to “Extremely.” High scores indicate better mood states in four domains. Many previous studies [20,23,24] used evaluation of subjective perceptions similar to this scale. The subjects of previous studies were patients with mild to moderate dementia who were able to communicate. The TDMS directly reflected the subjective perceptions of emotions of patients with dementia, despite the potential risk of self-report bias. The perception of the patients with dementia differed from that of the caregiver, so the observed assessment of the caregiver may not be accurate [25,26]. Self-reports of emotions in patients with dementia could be the most desirable source of data, as they are relatively unaffected by the dementia process [27].

### 2.4. Statistical Analysis

First, the Wilcoxon signed-rank test was used to compare scores before and after participants’ first session. Second, the scores of patients who participated multiple times were applied to the average scores. The average scores before and after each session were compared using the Wilcoxon signed-rank test to support the reliability of the first analysis. Because a single measurement is unreliable and may cause a measurement error, an average of multiple measurements was used to reduce the measurement error [28,29]. The average value of multiple measurements gave high reliability [30]. Two-sided significance tests were performed in the analyses. The significance level for the primary and secondary outcomes was set at *p* < 0.05. Data were analyzed using the Japanese version of SPSS for Windows version 20.0 (IBM Corporation, Armonk, NY, USA).

## 3. Results

Table 1 shows patients’ characteristics. Only 32 of the 33 participants were included in the first analysis due to missing data for one participant. Table 2 shows the results of mood scores before and after the first session. The primary outcome (pleasure) and one of the secondary outcomes (vitality) showed significant increases. However, other secondary outcomes (arousal and stability) did not show significant differences. Table 3 shows the results of average mood scores before and after multiple sessions. Only 22 of the 32 participants in the first analysis were included for the second analysis as they had participated in more than one session. There was a significant increase in the primary outcome (pleasure) after the sessions. However, other secondary outcomes (vitality, arousal, and stability) did not show significant differences.

## 4. Discussion

This retrospective single-arm cohort study investigated the effect of one-session CS intervention on the mood of patients with dementia in a hospital. The comparisons before and after participants’ first sessions showed two positive changes in the primary outcome (pleasure) and one of the secondary outcomes (vitality), but no changes in the arousal and stability scores (Table 2). In addition, the average score before and after multiple sessions showed a positive change in the primary outcome (pleasure), but no changes in the three secondary outcomes (Table 3), supporting the findings of the first analysis. Both the analyses showed positive changes in the outcome of pleasure. These results suggest that using a one-session CS intervention in a hospital setting could effectively improve pleasure among hospitalized patients with dementia.

The first analysis showed the improvement of the vitality score. The vitality score in TDMS consisted of two axes (high arousal/pleasure–low arousal/displeasure). The arousal score was not changed in this result; thus, improvement in the vitality score was greatly affected by the improved pleasure score. In addition, at the end of the 7-week CS therapy, Yamanaka et al. [22] used a face scale to assess mood improvement. Face scale improvement is considered a pleasant condition [31]. The study showed that the face scale score of the intervention group significantly improved compared with that of the control group. The effect size was observed as medium (0.30) in the mood difference between the intervention and control groups. Although this single-arm cohort study investigated the difference between before and after session scores, the effect size in the first analysis was observed as medium for pleasure (0.39), as seen in the previous study.

We believe that mood improvement using CS intervention was caused by the social interaction effect. In general, group psychotherapy is recommended for elderly people to relieve loneliness [32]. Patients with dementia who underwent group activity known as brain-activating rehabilitation in a hospital showed improved social interaction compared with the control group [33]. Bailey et al. [34] reported that group CS therapy had social benefits for patients with dementia. In qualitative interviews, Spector A et al. [35] reported that the experience in group CS therapy caused positive mood changes for patients with dementia. Conversely, individual CS therapy had no effect on quality of life [36].

This study provides preliminary evidence on the clinical efficacy of CS intervention. Studies are routinely performed in the early phases of clinical research to provide preliminary evidence in many clinical areas. These are designed to assess the safety and efficacy of interventions. They are commonly known as “feasibility” or “vanguard” studies [37]. Regarding the safety of this CS intervention study, no negative changes in the mood score (Table 2 and Table 3) and no adverse events (e.g., falls or worsening illness) were observed. Regarding the efficacy of this intervention, participants showed positive changes in pleasure. For this study, we also stratified the subjects according to dementia types and analyzed mood changes. The results of this analysis indicated that the effect size of the pleasure increase was large (0.62) in patients with Alzheimer’s disease (Table S1). Although the sample size was very small, this result suggests that CS interventions may be suitable for hospitalized patients with Alzheimer’s disease. Serving as an early phase of clinical research for this topic, this study’s results highlight the safety, efficacy, and feasibility of one-session CS interventions. Although this study could not prove that the one-session CS intervention improved the moods of hospitalized patients with dementia, it did provide proof-of-concept evidence prior to robust evaluation of the intervention. We believe that this study is beneficial for future clinical trials in a CS intervention in hospitals.

CS intervention, which leads to mood improvement, can be used in hospitals in various countries and may be effective for the management of neuropsychiatric symptoms. It can be performed by non-specialist healthcare workers and requires very limited equipment [38]. CS therapy for patients with dementia had benefits for cognition and quality of life, and costs were not different between the CS therapy group and usual treatment group [39], showing that CS therapy may be more cost-effective than usual treatment. In addition, for the management of neuropsychiatric symptoms, reducing stress and keeping patients with dementia engaged in activities that match their interests and capabilities are recommended [40,41]. Neuropsychiatric symptoms for longer hospitalization times were important risk factors in patients with dementia [4].

This study has several limitations. First, this study lacks validated measures for mood changes in patients with dementia. Although addressing this issue is necessary, we believe this study is important. This study directly reflects the subjective perceptions of emotions of patients with dementia. It may be the most desirable source of data. Second, because the single-arm design lacked a control group, responses could be due to the efficacy of the treatment or a natural history of improvement. Therefore, it is important to consider this result as preliminary evidence of the efficacy of the treatment and the most practical evidence for studying an intervention using a control group [42]. Third, all patients were female, as only females were willing to participate in this voluntary CS intervention. The men chose not to participate. Therefore, it is unclear whether the CS intervention would have a similar impact on other populations, specifically hospitalized men with dementia. Consequently, it is necessary to study the effects of CS intervention in both genders. Fourth, this study was conducted in one hospital only. Therefore, this finding may not be applicable to all hospitals. Fifth, this study did not stratify the participants according to dementia types. Such stratification is important to evaluate the differences in the effectiveness of the CS intervention among patients with different types of dementia. These five limitations show that the results of this study should not be over-interpreted.

## 5. Conclusions

This study is the first report to provide preliminary evidence that one-session CS intervention may have beneficial alterations in the moods of patients with dementia in a hospital. The comparison of the primary outcomes in patients with dementia before and after sessions showed a positive change, which may indicate an immediate improvement of pleasure. These preliminary results will lead to high-quality research in the future and may indicate the effectiveness of non-pharmacotherapeutic intervention in patients with dementia in a hospital.

## Figures and Tables

**Table 1:** Demographics of the study participants (n = 33).

Age, year	85.8 ± 7.8
MMSE	17.3 ± 5.0
Diagnosis of dementia	
AD	11 (33.3)
DLB	5 (15.2)
VD	1 (3)
Unspecified dementia	16 (48.5)
Diagnosis of hospitalization	
Orthopedic disease	20 (66.7)
Cerebrovascular disease	5 (16.7)
Respiratory disease	4 (13.3)
Urological diseases	2 (6.7)
Cardiovascular disease	1 (3.3)
Digestive disease	1 (3.3)

Notes: Values are means ± SD or numbers with percentages in parentheses. Abbreviations: MMSE, Mini-Mental State Examination; AD, Alzheimer’s disease; DLB, dementia with Lewy bodies; VD, vascular dementia.

**Table 2:** Results of mood scores before and after the first session (n = 32).

	Before Session		After Session			
	Median	Range	Median	Range	*p*	r
Pleasure	6	(−3 to 14)	8	(−6 to 18)	0.026 *	0.39
Arousal	−3	(−10 to 9)	−3	(−9 to 6)	0.398	0.15
Vitality	1	(−6 to 8)	2	(−4 to 9)	0.018 *	0.42
Stability	4.5	(−3 to 10)	6	(−4 to 10)	0.315	0.18

Abbreviations: *, *p* < 0.05.

**Table 3:** Results of average mood scores before and after multiple sessions (n = 22).

	Before Session		After Session			
	Median	Range	Median	Range	*p*	r
Pleasure	5.8	(−1.3 to 15.3)	7.8	(−1 to 17.4)	0.015 *	0.52
Arousal	−2.8	(−6.4 to 2.7)	−2.8	(−9 to 4.3)	0.299	0.22
Vitality	1	(−0.6 to 7.9)	2.1	(−3 to 8)	0.060	0.40
Stability	5.2	(−2 to 9)	5.6	(−2.7 to 9.7)	0.093	0.36

Abbreviations: *, *p* < 0.05.

## Data Availability

Data were not publicly deposited. However, we can share data and analyses that underpin the findings reported in this study. These are available on request from tkenji@gunma-u.ac.jp.

## Supplementary Materials

The following supporting information can be downloaded at: https://www.mdpi.com/article/10.3390/ijerph19031431/s1, Table S1 Results of mood scores before and after the first session for each dementia type.
