# Supplementary material for: Performing One-Session Cognitive Stimulation to Interact with Patients with Dementia in a Hospital for Mood Improvement: A Retrospective Single-Arm Cohort Study
Source: Int J Environ Res Public Health. 2022 Jan 27;19(3):1431. doi: 10.3390/ijerph19031431 (PMC8835672; doi:10.3390/ijerph19031431)
Supplement: Supplementary file 1 [file ijerph-19-01431-s001.zip › ijerph-1527180-supplementary.pdf]

Table S1. Results of mood scores before and after the first session for each dementia type

	Before session		After session			
	Median	Range	Median	Range	*p*	r
Pleasure						
AD (n=10)	5	(-3 to 14)	9	(-1 to 17)	0.052	0.62
DLB (n=5)	6	(-3 to 14)	3	(-1 to 17)	0.461	0.33
VD (n=1)	6		18			
Unspecified dementia (n=16)	6	(-3 to 14)	9	(-1 to 17)	0.117	0.39
Arousal						
AD (n=10)	-1.5	(-10 to 9)	-3	(-8 to 4)	0.953	0.02
DLB (n=5)	-3	(-10 to 9)	-4	(-8 to 4)	0.705	0.17
VD (n=1)	-6		0			
Unspecified dementia (n=16)	-3.5	(-10 to 9)	-3	(-8 to 4)	0.608	0.13
Vitality						
AD (n=10)	1.5	(-6 to 8)	4	(-1 to 8)	0.084	0.55
DLB (n=5)	2	(-6 to 8)	1	(-1 to 8)	1.000	0.00
VD (n=1)	0		9			
Unspecified dementia (n=16)	1	(-6 to 8)	2	(-1 to 8)	0.092	0.42
Stability						
AD (n=10)	3	(2 to 10)	6	(-2 to 10)	0.134	0.47
DLB (n=5)	4	(2 to 10)	2	(-2 to 10)	0.334	0.43
VD (n=1)	6		9			
Unspecified dementia (n=16)	5.5	(2 to 10)	7	(-2 to 10)	0.909	0.03

Abbreviations: AD = Alzheimer's disease; DLB = dementia with Lewy bodies; VD = vascular dementia.
